# Daily Intake of Sugar-Sweetened Beverages Among US Adults in 9 States, by State and Sociodemographic and Behavioral Characteristics, 2016

**DOI:** 10.5888/pcd15.180335

**Published:** 2018-12-13

**Authors:** Elizabeth A. Lundeen, Sohyun Park, Liping Pan, Heidi M. Blanck

**Affiliations:** 1 Division of Nutrition, Physical Activity and Obesity, National Center for Chronic Disease Prevention and Health Promotion, Centers for Disease Control and Prevention, Atlanta, Georgia

## Abstract

We examined associations between sugar-sweetened beverage (SSB) intake — a chronic disease risk factor — and characteristics of 75,029 adults (≥18 y) in 9 states by using 2016 Behavioral Risk Factor Surveillance System (BRFSS) data. We used multinomial logistic regression to estimate adjusted odds ratios for SSB intake categorized as none (reference), fewer than 1 time per day, and 1 or more times per day, by sociodemographic and behavioral characteristics. Overall, 32.1% of respondents drank SSBs 1 or more times per day. We found higher odds for 1 or more times per day among younger respondents, men, Hispanic and non-Hispanic black respondents, current smokers, respondents residing in nonmetropolitan counties, employed respondents, and those with less than high school education, obesity, and no physical activity. Our findings can inform the targeting of efforts to reduce SSB consumption.

## Objective

Sugar-sweetened beverages (SSBs) — nondiet soda, fruit drinks that are not 100% juice, sweet tea, sports drinks, and energy drinks — are the largest source of added sugars in the diet of US adults (1). National Health and Nutrition Examination Survey data showed that 49.3% of US adults consumed 1 or more SSBs on a given day during 2011–2014 (2). Frequent SSB consumption is associated with increased risk of weight gain, type 2 diabetes, hypertension, asthma, and dental caries (3–6). The objective of this study was to update data for 9 states on the frequency of SSB intake and its association with selected sociodemographic and behavioral characteristics.

## Methods

We analyzed 2016 data from the Behavioral Risk Factor Surveillance System (BRFSS), a state-based random-digit–dial telephone survey of US adults, conducted annually to monitor health conditions and behaviors. BRFSS uses multistage, stratified sampling to select a representative sample of noninstitutionalized adults in each state, the District of Columbia, and selected US territories. Nine states included questions from the SSB optional module in 2016: Delaware, Indiana, Iowa, Mississippi, New Jersey, New York, Ohio, Texas, and West Virginia. These states had a median combined landline and cell phone response rate of 43.0% (range, 36.3%–55.2%) (7). SSB questions were 1) “During the past 30 days, how often did you drink regular soda/pop containing sugar? Do not include diet soda/pop.” and 2) “During the past 30 days, how often did you drink sugar-sweetened fruit drinks (eg, Kool-Aid/lemonade), sweet tea, and sports or energy drinks (eg, Gatorade/Red Bull)? Do not include 100% fruit juice, diet drinks, or artificially sweetened drinks.” We converted SSB consumption, reported as number of times per day, week, or month, to daily intake for both questions and then summed to calculate daily SSB intake, categorized as none, more than 0 to fewer than 1 time per day (labeled as <1 time per day), and 1 or more times per day (8). We excluded respondents who were missing SSB data (n = 10,731 [12.5%]), leaving 75,029 adults. Compared with respondents who did not have SSB data, respondents who had SSB data were older, had more education, and were more likely to be non-Hispanic white or female. We used χ^2^ tests to examine whether frequency of SSB intake differed by population subgroup (significant at *P* < .05). We performed exploratory multinomial logistic regression, using complete case analysis (n = 67,965) and complex survey design sampling weights, to estimate adjusted odds ratios (AORs) for SSB intake of fewer than 1 time per day and 1 or more times per day (reference: none), by all characteristics in the model: state, age, sex, race/ethnicity, education, marital status, employment status, weight status, smoking status, alcohol intake, leisure-time physical activity, and metropolitan status (residing in a metropolitan county or nonmetropolitan county [9]).

## Results

Of the 75,029 respondents in 9 states, 47.9% were men and 52.1% were women (Table 1). Most were non-Hispanic white (62.8%), married (51.5%), and employed (57.1%) and had completed at least some college or technical school (57.1%). SSBs were never consumed by 26.4% of respondents, consumed fewer than 1 time per day by 41.5%, and consumed 1 or more times per day by 32.1%. SSB intake of more than 2 times per day was reported by 17.1% of respondents. Frequency of SSB consumption 1 or more times per day ranged from 21.5% in New Jersey to 46.8% in Mississippi.

**Table 1:** Frequency of Sugar-Sweetened Beverage^a^ Intake, by Sociodemographic and Behavioral Characteristics, Among Adults Aged ≥18 in 9 States, Behavioral Risk Factor Surveillance System, 2016

Characteristic	Total, n (%)^b^	Sugar-Sweetened Beverage Intake, % (95% Confidence Interval)^c^		
		None	<1 Time per Day	≥1 Time per Day
**Total sample**	75,029 (100.0)	26.4 (25.5–27.3)	41.5 (40.4–42.6)	32.1 (31.0–33.2)
**States (n = 75,029)**				
Delaware	3,565 (1.2)	29.4 (27.5–31.4)	39.6 (37.3–41.9)	31.0 (28.9–33.2)
Indiana	9,744 (7.9)	22.5 (21.5–23.6)	39.4 (38.0–40.8)	38.1 (36.6–39.6)
Iowa	6,296 (3.8)	27.7 (26.4–29.0)	43.1 (41.5–44.7)	29.2 (27.7–30.8)
Mississippi	4,549 (3.6)	19.1 (17.7–20.6)	34.1 (32.2–36.0)	46.8 (44.8–48.8)
New Jersey	6,464 (10.3)	37.0 (35.2–38.9)	41.5 (39.5–43.4)	21.5 (19.9–23.3)
New York	29,547 (22.9)	32.3 (31.3–33.4)	44.3 (43.1–45.6)	23.3 (22.3–24.4)
Ohio	3,452 (15.1)	25.2 (23.2–27.2)	40.7 (38.2–43.2)	34.2 (31.7–36.7)
Texas	4,773 (32.8)	21.4 (19.2–23.8)	41.3 (38.4–44.3)	37.3 (34.3–40.3)
West Virginia	6,639 (2.5)	21.3 (20.2–22.4)	38.4 (37.0–39.8)	40.4 (38.9–41.8)
**Age group, y (n = 75,029)**				
18–24	3,475 (12.3)	9.9 (7.4–13.0)	46.7 (42.4–51.0)	43.4 (39.2–47.8)
25–34	6,895 (17.0)	13.7 (11.8–15.9)	45.2 (42.0–48.4)	41.1 (37.9–44.4)
35–44	8,208 (16.6)	18.8 (16.7–20.9)	43.5 (40.6–46.5)	37.7 (34.8–40.8)
45–54	12,183 (17.3)	28.7 (26.5–30.9)	40.4 (38.0–42.8)	30.9 (28.5–33.4)
≥55	44,268 (36.9)	40.1 (38.9–41.4)	37.7 (36.4–39.0)	22.2 (21.0–23.4)
**Sex (n = 75,029)**				
Male	31,802 (47.9)	22.4 (21.1–23.8)	42.1 (40.5–43.7)	35.5 (33.9–37.1)
Female	43,227 (52.1)	30.1 (28.9–31.3)	40.9 (39.4–42.5)	29.0 (27.4–30.6)
**Race/ethnicity (n = 73,740)**				
Non-Hispanic white	60,679 (62.8)	30.2 (29.2–31.2)	39.3 (38.1–40.4)	30.5 (29.3–31.8)
Non-Hispanic black	5,708 (12.1)	18.5 (15.7–21.5)	44.3 (40.7–48.0)	37.2 (33.7–40.9)
Hispanic	4,689 (18.5)	18.3 (16.1–20.6)	44.1 (40.8–47.5)	37.6 (34.3–41.1)
Non-Hispanic other	2,664 (6.6)	26.2 (21.9–31.1)	51.0 (45.8–56.1)	22.8 (19.1–27.0)
**Education (n = 74,820)**				
Some high school	6,353 (14.2)	22.0 (19.4–24.9)	33.8 (30.7–37.1)	44.2 (40.5–47.9)
High school graduate or GED	22,865 (28.7)	21.9 (20.6–23.2)	38.8 (36.8–40.7)	39.4 (37.4–41.4)
Some college or technical school	19,494 (30.4)	26.7 (24.8–28.6)	41.9 (39.7–44.2)	31.4 (29.3–33.6)
College graduate	26,108 (26.7)	33.2 (31.7–34.7)	48.0 (46.3–49.8)	18.8 (17.3–20.3)
**Marital status (n = 74,627)**				
Married	38,400 (51.5)	30.0 (28.7–31.2)	42.2 (40.7–43.6)	27.9 (26.4–29.3)
Single	13,671 (28.4)	16.7 (15.0–18.5)	43.5 (41.1–45.9)	39.8 (37.4–42.2)
Divorced/separated/widowed	22,556 (20.1)	30.9 (29.1–32.8)	37.1 (34.9–39.3)	32.0 (29.6–34.4)
**Employment status (n = 74,554)**				
Employed	35,858 (57.1)	22.9 (21.8–24.1)	42.5 (41.0–44.1)	34.5 (33.0–36.1)
Not employed	15,134 (25.4)	23.1 (21.2–25.1)	41.4 (39.1–43.8)	35.5 (33.2–37.9)
Retired	23,562 (17.5)	42.5 (40.7–44.3)	37.9 (36.2–39.8)	19.5 (18.1–21.0)
**Weight status^d^ (n = 70,611)**				
Underweight/normal weight	22,269 (32.5)	28.4 (26.8–30.0)	41.4 (39.5–43.3)	30.2 (28.4–32.1)
Overweight	25,324 (35.1)	26.9 (25.4–28.5)	42.5 (40.6–44.4)	30.6 (28.8–32.4)
Obesity	23,018 (32.4)	23.2 (21.6–24.8)	39.9 (37.9–42.0)	36.9 (34.7–39.2)
**Smoking status (n = 74,651)**				
Nonsmoker	40,591 (59.4)	26.2 (25.1–27.5)	45.1 (43.6–46.6)	28.7 (27.2–30.2)
Former smoker	21,959 (24.1)	33.4 (31.5–35.3)	39.5 (37.4–41.6)	27.1 (25.1–29.2)
Current smoker	12,101 (16.5)	16.8 (15.2–18.5)	31.6 (29.2–34.1)	51.6 (49.0–54.2)
**Alcohol intake^e^ (n=74,361)**				
None	36,481 (47.2)	26.9 (25.6–28.2)	38.4 (36.8–40.0)	34.7 (33.1–36.4)
Any	33,797 (46.8)	25.7 (24.4–27.0)	44.7 (43.1–46.3)	29.6 (28.1–31.3)
Heavy	4,083 (6.0)	26.4 (22.5–30.8)	41.9 (37.3–46.6)	31.7 (27.3–36.5)
**Leisure-time physical activity^f^ (n = 74,925)**				
Participated in physical activity or exercise	54,147 (73.6)	26.9 (25.9–27.9)	43.4 (42.1–44.7)	29.7 (28.4–31.0)
Did not participate in physical activity or exercise	20,778 (26.4)	25.0 (23.4–26.8)	36.2 (34.2–38.2)	38.8 (36.6–41.0)
**Metropolitan status (n = 75,029)**				
Metropolitan county	51,149 (83.0)	27.4 (26.4–28.4)	42.0 (40.7–43.2)	30.6 (29.4–31.9)
Nonmetropolitan county	23,880 (17.0)	21.5 (19.8–23.3)	39.2 (36.7–41.7)	39.3 (36.6–42.1)

a Includes nondiet soda, fruit drinks that are not 100% juice, sweet tea, sports drinks, and energy drinks.

b Unweighted sample size and weighted percentage. Weighted percentages may not add to 100 because of rounding.

c χ^2^ tests were used for each variable to examine differences across categories. All *P* values <.001.

d Classified based on body mass index (BMI): underweight/normal weight (BMI <25.0 kg/m^2^), overweight (BMI 25.0 to <30.0 kg/m^2^), obesity (BMI ≥30.0 kg/m^2^).

e Alcohol intake was categorized as none, any (≥1 drink of any alcoholic beverage during the past month but not heavy drinking), and heavy (>2 drinks per day for men and >1 drink per day for women).

f Leisure-time physical activity was categorized as 1) participating in any or 2) not participating in any physical activity or exercise during the past 30 days other than in a regular job.

SSB intake differed by state, age, sex, race/ethnicity, education, marital status, employment status, weight status, smoking status, alcohol intake, leisure-time physical activity, and metropolitan status (all *P* < .001 by χ^2^ test). The prevalence of SSB intake of 1 or more times per day was higher among men (35.5%) than among women. By age group, it was highest among respondents aged 18 to 24 years (43.4%); by race/ethnicity, it was highest among Hispanic respondents (37.6%) and non-Hispanic black respondents (37.2%); by education, highest among those with some high school education (44.2%); by marital status, highest among respondents who were single (39.8%); by employment status, highest among respondents not employed (35.5%); by smoking status, highest among current smokers (51.6%); by weight status, highest among respondents with obesity (36.9%); by metropolitan status, higher among residents of nonmetropolitan counties (39.3%); by alcohol intake, highest among respondents who consumed no alcohol in the past month (34.7%); and by leisure-time physical activity, higher among respondents who had no physical activity in the past month (38.8%).

Adjusted odds of drinking SSBs 1 or more times per day were significantly higher among respondents aged 18 to 54 (AOR range, 1.82–7.84) versus those aged 55 or older; men (AOR = 1.66) versus women; Hispanic (AOR = 1.43) and non-Hispanic black respondents (AOR = 1.65) versus non-Hispanic white respondents; respondents who did not complete college (AOR range,1.67–2.71) versus those who completed college; respondents who were employed (AOR = 1.30) versus those not employed; respondents with obesity (AOR = 1.40) versus those who were underweight/normal weight; current smokers (AOR = 2.44) versus nonsmokers; respondents who did not participate physical activity in the past month (AOR = 1.39) versus those who did participate; and residents of nonmetropolitan counties (AOR = 1.36) versus metropolitan counties (Table 2). Odds for SSB intake of 1 or more times per day were significantly lower among heavy alcohol drinkers (AOR = 0.65) versus those with no alcohol consumption in the past month. We observed somewhat similar patterns among respondents who consumed SSBs fewer than 1 time per day.

**Table 2:** Adjusted Odds Ratios for Sugar-Sweetened Beverage Intake ≥1 Time per Day and <1 Time per Day, by Sociodemographic and Behavioral Characteristics Among Adults in 9 States, Behavioral Risk Factor Surveillance System, 2016

Characteristic	Sugar-Sweetened Beverage^a^ Intake, Adjusted Odds Ratio^b^ (95% Confidence Interval)	
	≥1 Time per Day	<1 Time per Day
**State**		
Delaware	1.49 (1.28–1.74)	1.05 (0.92–1.20)
Indiana	2.28 (2.02–2.57)	1.42 (1.28–1.57)
Iowa	1.38 (1.20–1.59)	1.18 (1.05–1.32)
Mississippi	2.69 (2.26–3.20)	1.27 (1.09–1.49)
New Jersey	0.84 (0.73–0.97)	0.86 (0.77–0.97)
New York^c^	1.00 [Reference]	1.00 [Reference]
Ohio	1.81 (1.52–2.15)	1.28 (1.10–1.48)
Texas	2.16 (1.76–2.66)	1.22 (1.02–1.47)
West Virginia	2.40 (2.09–2.76)	1.52 (1.35–1.72)
**Age group, y**		
18–24	7.84 (5.11–12.03)	4.67 (3.17–6.90)
25–34	5.13 (3.96–6.65)	3.15 (2.50–3.97)
35–44	3.48 (2.77–4.37)	2.12 (1.75–2.58)
45–54	1.82 (1.50–2.21)	1.41 (1.20–1.65)
≥55	1.00 [Reference]	1.00 [Reference]
**Sex**		
Male	1.66 (1.44–1.90)	1.35 (1.20–1.51)
Female	1.00 [Reference]	1.00 [Reference]
**Race/ethnicity**		
Non-Hispanic white	1.00 [Reference]	1.00 [Reference]
Non-Hispanic black	1.65 (1.25–2.18)	1.70 (1.33–2.17)
Hispanic	1.43 (1.12–1.84)	1.72 (1.38–2.14)
Other non-Hispanic	0.77 (0.54–1.11)	1.33 (0.98–1.80)
**Education**		
Some high school	2.38 (1.82–3.12)	0.88 (0.70–1.12)
High school graduate or GED	2.71 (2.29–3.21)	1.25 (1.09–1.43)
Some college or technical school	1.67 (1.39–2.01)	1.02 (0.88–1.18)
College graduate	1.00 [Reference]	1.00 [Reference]
**Marital status**		
Married	1.00 [Reference]	1.00 [Reference]
Single	1.03 (0.83–1.27)	0.95 (0.79–1.14)
Divorced/separated/widowed	1.14 (0.97–1.34)	1.05 (0.92–1.19)
**Employment status**		
Employed	1.30 (1.07–1.57)	1.08 (0.92–1.27)
Not employed	1.00 [Reference]	1.00 [Reference]
Retired	0.84 (0.69–1.04)	0.93 (0.78–1.10)
**Weight status^d^**		
Underweight/normal weight	1.00 [Reference]	1.00 [Reference]
Overweight	1.06 (0.90–1.25)	1.14 (1.0–1.30)
Obesity	1.40 (1.18–1.68)	1.23 (1.07–1.43)
**Smoking status**		
Nonsmoker	1.00 [Reference]	1.00 [Reference]
Former smoker	0.94 (0.80–1.10)	0.87 (0.76–0.99)
Current smoker	2.44 (2.06–2.91)	1.13 (0.96–1.34)
**Alcohol intake^e^**		
None	1.00 [Reference]	1.00 [Reference]
Any	0.89 (0.76–1.03)	1.15 (1.01–1.29)
Heavy	0.65 (0.49–0.88)	0.94 (0.72–1.23)
**Leisure-time physical activity^f^**		
Participated in physical activity or exercise	1.00 [Reference]	1.00 [Reference]
Did not participate in physical activity or exercise	1.39 (1.20–1.61)	1.02 (0.89–1.16)
**Metropolitan status**		
Metropolitan county	1.00 [Reference]	1.00 [Reference]
Nonmetropolitan county	1.36 (1.15–1.61)	1.25 (1.08–1.44)

a Includes nondiet soda, fruit drinks that are not 100% juice, sweet tea, sports drinks, and energy drinks.

b A multinomial logistic regression model was used to calculate adjusted odds ratios for adult sugar-sweetened beverage intake <1 time per day and ≥1 time per day (reference: none). The model contained all sociodemographic and behavioral characteristics and included 67,965 adults with data for all variables studied.

c New York was chosen as the reference because this state had the largest sample size.

d Classified according to body mass index (BMI): underweight/normal weight (BMI <25.0 kg/m^2^), overweight (BMI 25.0 to <30.0 kg/m^2^), obesity (BMI ≥30.0 kg/m^2^).

e Alcohol intake was categorized as none, any (≥1 drink of any alcoholic beverage during the past month but not heavy drinking), and heavy (>2 drinks per day for men and >1 drink per day for women).

f Leisure-time physical activity was categorized as 1) participating in any or 2) not participating in any physical activity or exercise during the past 30 days other than in a regular job.

## Discussion

Daily SSB consumption among adults is high in the United States and varies across states and subgroups. Among 9 states surveyed in 2016, nearly 1 in 3 adults consumed SSBs 1 or more times per day. In the 2013 BRFSS, which included 23 states and the District of Columbia, 29.1% of respondents were daily consumers (10). Our findings were consistent with findings of other studies documenting higher SSB intake among younger adults, males, non-Hispanic blacks, adults with less education, current smokers, and those who are physically inactive (10,11).

We found that respondents with obesity had significantly higher odds of consuming SSBs 1 or more times per day than underweight or normal-weight respondents. Longitudinal studies have documented the association between daily SSB intake and weight gain (3,4). Previous analyses of BRFSS SSB data did not explore associations with metropolitan status. Our finding that SSB intake was higher in nonmetropolitan (eg, rural) counties than in metropolitan (eg, urban) counties is novel. A 2006 study in Texas reported higher prevalence of any SSB intake and high SSB intake among rural adults than among urban adults; among rural adults, high SSB intake (≥3 cans or glasses of SSBs per day) was associated with poverty, food insecurity, and fast food consumption (12).

Our study has several limitations. BRFSS data are self-reported; however, potential bias in SSB intake data related to self-report or social desirability is not known. Our analysis included only 9 states that asked SSB questions; thus, the findings might not be generalizable to other states. Finally, the amount or calorie intake from SSBs cannot be estimated, because data on SSB intake was captured as frequency rather than volume. Our findings that adult SSB intake varied across states and certain characteristics can be used to help inform efforts to reduce SSB consumption.

## References

[1] Drewnowski A , Rehm CD . Consumption of added sugars among US children and adults by food purchase location and food source. Am J Clin Nutr 2014;100(3):901–7. 10.3945/ajcn.114.089458 25030785 PMC4135498

[2] Rosinger A , Herrick K , Gahche J , Park S . Sugar-sweetened beverage consumption among US adults, 2011–2014. NCHS Data Brief 2017;270(270):1–8. 28135185

[3] Schulze MB , Manson JE , Ludwig DS , Colditz GA , Stampfer MJ , Willett WC , Sugar-sweetened beverages, weight gain, and incidence of type 2 diabetes in young and middle-aged women. JAMA 2004;292(8):927–34. 10.1001/jama.292.8.927 15328324

[4] Dhingra R , Sullivan L , Jacques PF , Wang TJ , Fox CS , Meigs JB , Soft drink consumption and risk of developing cardiometabolic risk factors and the metabolic syndrome in middle-aged adults in the community. Circulation 2007;116(5):480–8. 10.1161/CIRCULATIONAHA.107.689935 17646581

[5] Park S , Akinbami LJ , McGuire LC , Blanck HM . Association of sugar-sweetened beverage intake frequency and asthma among US adults, 2013. Prev Med 2016;91:58–61. 10.1016/j.ypmed.2016.08.004 27496394 PMC5050122

[6] Bernabé E , Vehkalahti MM , Sheiham A , Aromaa A , Suominen AL . Sugar-sweetened beverages and dental caries in adults: a 4-year prospective study. J Dent 2014;42(8):952–8. 10.1016/j.jdent.2014.04.011 24813370

[7] Centers for Disease Control and Prevention. BRFSS combined landline and cell phone weighted response rates by state, 2016. https://www.cdc.gov/brfss/annual_data/2016/pdf/2016_ResponseRates_Table.pdf.

[8] Park S , Pan L . A data user’s guide to the BRFSS sugar-sweetened beverage questions: how to analyze consumption of sugar-sweetened beverages. https://www.cdc.gov/brfss/data_documentation/pdf/brfss_ssb-userguide.pdf.

[9] Centers for Disease Control and Prevention. NCHS urban-rural classification scheme for counties. https://www.cdc.gov/nchs/data_access/urban_rural.htm.

[10] Park S , Xu F , Town M , Blanck HM . Prevalence of sugar-sweetened beverage intake among adults — 23 states and the District of Columbia, 2013. MMWR Morb Mortal Wkly Rep 2016;65(7):169–74. 10.15585/mmwr.mm6507a1 26914018

[11] Park S , Pan L , Sherry B , Blanck HM . Consumption of sugar-sweetened beverages among US adults in 6 states: Behavioral Risk Factor Surveillance System, 2011. Prev Chronic Dis 2014;11:E65. 10.5888/pcd11.130304 24762529 PMC4008944

[12] Sharkey JR , Johnson CM , Dean WR . Less-healthy eating behaviors have a greater association with a high level of sugar-sweetened beverage consumption among rural adults than among urban adults. Food Nutr Res 2011;55(1):1–6. 10.3402/fnr.v55i0.5819 21845142 PMC3153312

